# Anesthetics affect peripheral venous pressure waveforms and the cross-talk with arterial pressure

**DOI:** 10.1007/s10877-020-00632-6

**Published:** 2021-02-19

**Authors:** Ali Z. Al-Alawi, Kaylee R. Henry, Lauren D. Crimmins, Patrick C. Bonasso, Md Abul Hayat, Melvin S. Dassinger, Jeffrey M. Burford, Hanna K. Jensen, Joseph Sanford, Jingxian Wu, Kevin W. Sexton, Morten O. Jensen

**Affiliations:** 1 Department of Biomedical Engineering, University of Arkansas, Fayetteville, AR USA; 2 Division of Pediatric Surgery, University of Arkansas for Medical Sciences, Little Rock, AR USA; 3 Department of Electrical Engineering, University of Arkansas, Fayetteville, AR USA; 4 Department of Anesthesiology, University of Arkansas for Medical Sciences, Little Rock, AR USA; 5 Department of Surgery, University of Arkansas for Medical Sciences, Little Rock, AR USA

**Keywords:** Peripheral venous pressure, Central venous pressure, Inhaled anesthetic, Infused anesthetic, Cross-talk between peripheral and arterial circulation

## Abstract

Analysis of peripheral venous pressure (PVP) waveforms is a novel method of monitoring intravascular volume. Two pediatric cohorts were studied to test the effect of anesthetic agents on the PVP waveform and cross-talk between peripheral veins and arteries: (1) dehydration setting in a pyloromyotomy using the infused anesthetic propofol and (2) hemorrhage setting during elective surgery for craniosynostosis with the inhaled anesthetic isoflurane. PVP waveforms were collected from 39 patients that received propofol and 9 that received isoflurane. A multiple analysis of variance test determined if anesthetics influence the PVP waveform. A prediction system was built using k-nearest neighbor (k-NN) to distinguish between: (1) PVP waveforms with and without propofol and (2) different minimum alveolar concentration (MAC) groups of isoflurane. 52 porcine, 5 propofol, and 7 isoflurane subjects were used to determine the cross-talk between veins and arteries at the heart and respiratory rate frequency during: (a) during and after bleeding with constant anesthesia, (b) before and after propofol, and (c) at each MAC value. PVP waveforms are influenced by anesthetics, determined by MANOVA: *p value* < 0.01, *η*^2^ = 0.478 for hypovolemic, and *η*^2^ = 0.388 for euvolemic conditions. The k-NN prediction models had 82% and 77% accuracy for detecting propofol and MAC, respectively. The cross-talk relationship at each stage was: (a) *ρ* = 0.95, (b) *ρ* = 0.96, and (c) could not be evaluated using this cohort. Future research should consider anesthetic agents when analyzing PVP waveforms developing future clinical monitoring technology that uses PVP.

**Electronic supplementary material:**

The online version of this article (10.1007/s10877-020-00632-6) contains supplementary material, which is available to authorized users.

## Introduction

During trauma or illness, dehydration can become life-threatening if it is not diagnosed and treated before organ damage occurs. Assessing dehydration can be complicated, as there is not a universal method for predicting the fluid volume status of a dehydrated child or adult, and often vital signs are unable to predict fluid loss before severe side effects become present [1]. Each year, over 30 million children are affected by dehydration, and 400,000 pediatric emergency room visits are due to dehydration [2, 3]. Additionally, the leading preventable cause of death in casualty care settings is hemorrhage [4, 5].

Analysis of peripheral venous pressure (PVP) waveforms is a novel method of monitoring intravascular volume, especially in cases of dehydration and hemorrhage [6, 7], and may provide earlier sensitivity in detection of loss of blood volume. Previously, Bonasso et al. demonstrated that PVP waveforms are more sensitive in predicting volume loss than vital signs in a large animal model of bleeding [8]. Furthermore, PVP has been shown to be a predictor of dehydration in a pediatric cohort who underwent operations for pyloric stenosis. However, PVP waveforms can potentially be confounded by parameters other than volume status, such as anesthetic agents, while collecting the data [8]. We hypothesize that infused and inhaled anesthetics have an impact on the PVP waveforms and tested this in two anesthetized patient cohorts. After determining the impact of anesthetics on the PVP waveforms, we also analyze how peripheral arterial and venous circulation impact each other before and after an anesthetic is applied. Currently, no other PVP signal analysis algorithms take into account the confounding factor of anesthetics or the heart rate of a patient, which is an important limitation of PVP research.

### Relationship between peripheral arterial and venous circulation

**Fig. 1:**
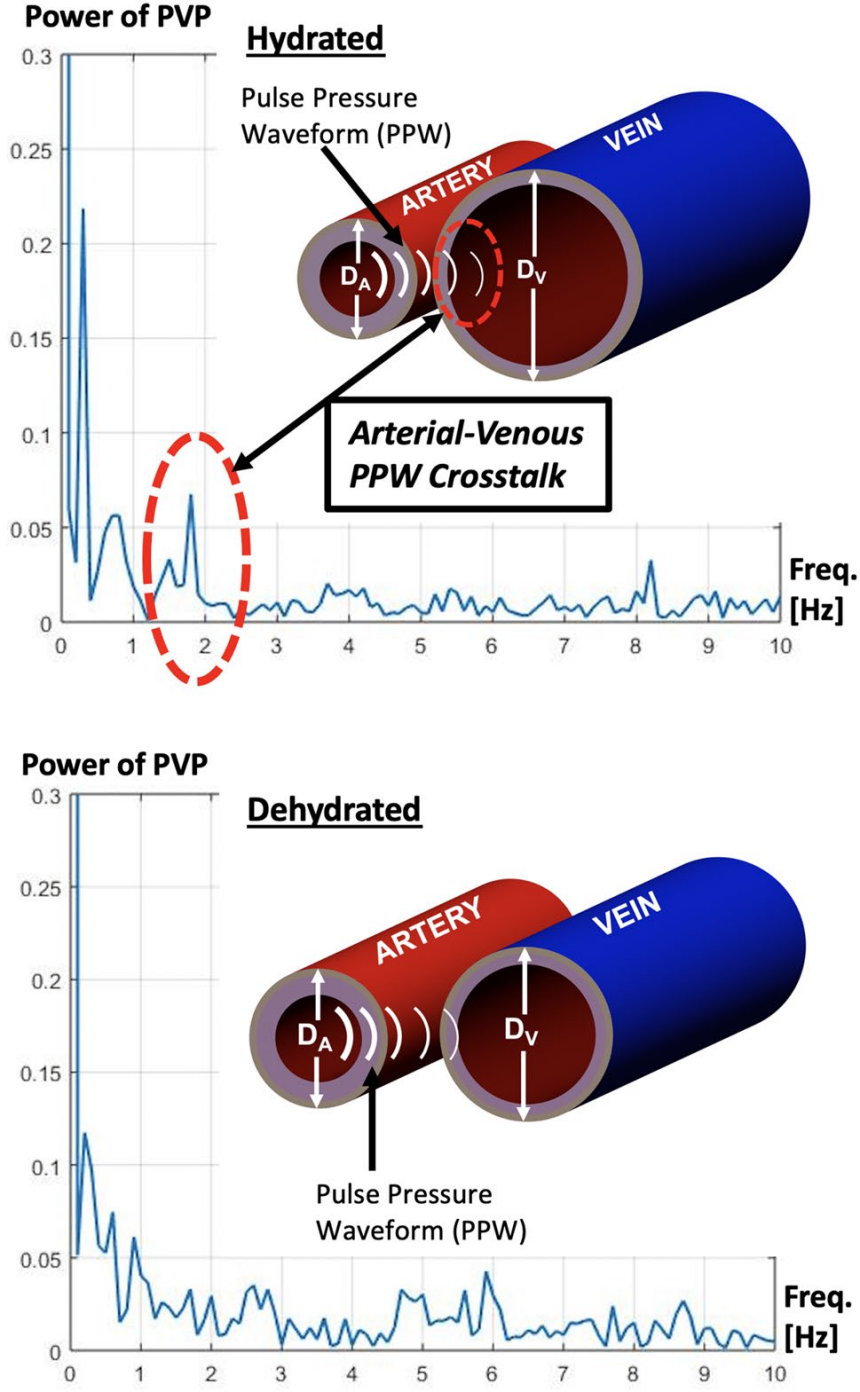
The power spectral density (PSD) of peripheral venous pressure (PVP) for the hydrated patient (top) identifies a peak at frequencies around the heart rate (red dotted line). In this example, the peak is shown at approximately 1.8 Hz = 108 bpm. In the same patient during dehydration (bottom), this phenomenon does not exist. The vein diameter (D_V_) is significantly larger in the hydrated state. The arterial diameter (D_A_) changes slightly between hydration and dehydration. Combined, this causes the hydromechanical interaction of pressure signals between the arterial and the venous side of the circulation to be stronger when the patient is hydrated. Reprinted with permission by Springer Nature from [16] Bonasso et al. “Optimizing peripheral venous pressure waveforms in an awake pediatric patient by decreasing signal interference”, Journal of Clinical Monitoring and Computing 2018

In human extremities, peripheral arteries and veins run in close anatomical proximity, and it is feasible to assume that the pressure in one vessel can potentially carry over to the other. Previous findings suggest the presence of this type of hydro-mechanical interaction between the arterial and venous circulation, known as “cross-talk”, in a cohort of dehydrated infants as depicted in Fig. 1 [8].

It appears as though in hydrated patients, the cross-talk between arteries and veins in direct physical interaction with each other accounts for the signal waveform frequencies corresponding to heart rate. When the patient has adequate blood volume, the arterial pulse pressure waveform crosses over to the venous side. In dehydrated patients, as the diameter of arteries and veins decreases, the cross-talk is absent; therefore, the signal waveform is affected at the frequency of the heart rate [8].

The term “cross-talk” and its presence due to mechanical properties of the veins and arteries was proposed in Bonasso et al., where they were able to show that the cross-talk was present due to the crossing over of the arterial pulse pressure waveform to the venous circulation [8]. The cross-talk was proposed as an additional mechanism to the relationship described in Alian et al., where it was shown that the venous pressure waveform reflected changes in pressure from the right atrium [9]. Since the cross-talk is due only to the pulse pressure waveform which comes from the aorta and travels through the arterial tree before mechanically impacting the surrounding tissues, only the mechanical stimulus of the arteries is considered. In fact, whenever a pulse is checked on the wrist using the index and middle finger, the mechanical effect of the cross-talk can actually be felt and is clearly detectable.

For this study, once an anesthetic has been applied and thus caused vasodilation of the vessels, it is expected that the cross-talk will be present because the peripheral arteries and veins will be in closer proximity to each other. The results are expected to be similar to the results from Bonasso et al. in Fig. 1 where the cross-talk became present once the patient became hydrated [8].

### Cohorts

The first cohort represented a dehydration setting in infants operated on for pyloric stenosis. Infants who had been projectile vomiting were diagnosed via ultrasound and admitted prior to undergoing a pyloromyotomy operation in which propofol was infused as an anesthetic [10, 11] . Data was collected after being resuscitated to near euvolemia at the time of operation.

The second cohort represented a controlled hemorrhage setting in infants affected by craniosynostosis, while undergoing an elective reconstructive craniotomy. Due to the vast blood supply to the skull and an intraoperative estimated blood loss of 60-70 cc/kg, up to half of the blood volume may need to be replaced utilizing a combination of intravenous fluids, blood products and occasionally vasopressors [12–14]. The depth of the patient’s anesthesia both in the hemorrhagic and nonhemorrhagic portion of the surgery is controlled by altering the minimum alveolar concentration (MAC) of isoflurane, where a higher MAC corresponds to a higher dosage of the anesthetic [15].

A third cohort represented a controlled hemorrhage setting in healthy porcine. PVP and electrocardiogram (EKG) signals were collected during a resting state before bleeding, and during the bleeding.

The first two cohorts were utilized to determine if anesthetics such as propofol or isoflurane influenced the PVP waveform. After determining the relationship, two machine learning systems were built using a k-nearest neighbor statistical model to predict hydration levels for arbitrary pyloric stenosis PVP waveforms, and also predict MAC for an arbitrary craniosynostosis PVP waveform. Then, all three cohorts were utilized to determine the relationship between peripheral venous and arterial circulation before and after bleeding for both before and after anesthetic conditions.

## Methods

### Acquiring pediatric PVP

**Fig. 2:**
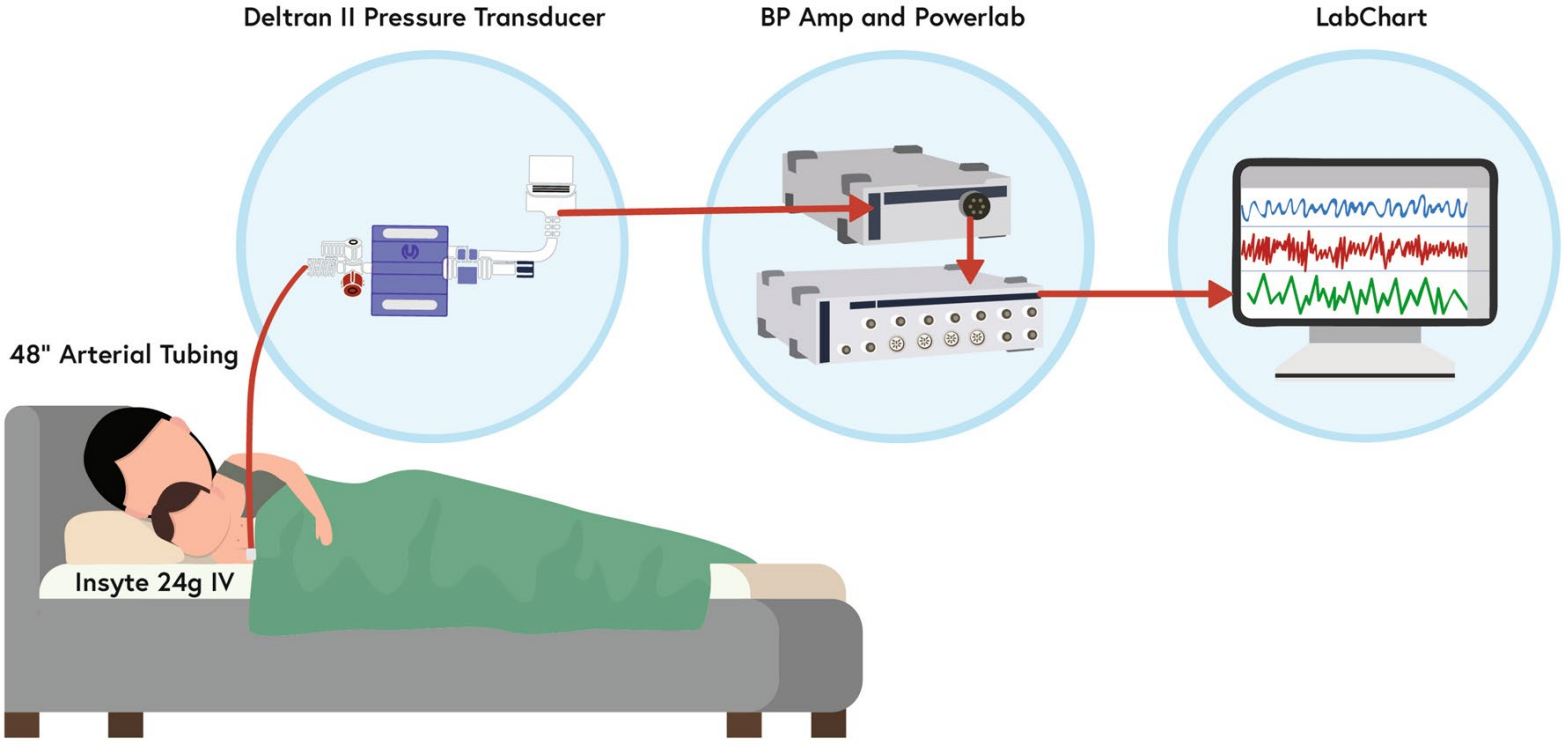
PVP waveform data acquisition setup consisting of 48″ arterial tubing (*Smiths Medical*), a Deltran II pressure transducer (*ADInstruments*), BP Amp and Powerlab (*ADInstruments*). Waveforms are viewed using the software LabChart (*ADInstruments*). Reprinted with permission by Elsevier from [8] Bonasso et al. “Venous Physiology Predicts Dehydration in the Pediatric Population”, Journal of Surgical Research, June 2019

This study was performed in accordance with the University of Arkansas for Medical Sciences Institutional Review Board, IRB #206193. Informed consent was obtained from all individual participants included in the study. PVP waveforms were collected from 39 pyloric stenosis patients and 9 craniosynostosis patients. For the pyloric stenosis patients, data points were collected over the entire operation, and for the craniosynostosis patients, data points were collected from the first instance of isoflurane throughout the procedure until isoflurane administration was ceased. PVP waveforms were measured with a 24-gauge Insyte-N Autoguard peripheral intravenous (PIV) catheter (*Becton Dickinson Infusion Therapy Systems, Sandy, Utah, USA*) at Arkansas Children’s Hospital. The PIV catheter was connected to a Deltran II pressure transducer (*ADInstruments, Colorado Springs, CO, USA*) using 48-in. arterial pressure tubing (*Smiths Medical, Dublin, Ohio, USA*). Then, a Powerlab data acquisition system (*ADInstruments*) was used to connect the hardware setup with LabChart 8 (*ADInstruments*) to record the waveforms. The acquisition setup is illustrated in Fig. 2. The Deltran pressure transducer detects small movements of the infant, which interferes with the PVP recording.

**Fig. 3:**
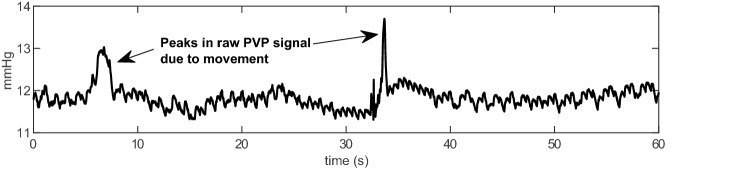
PVP waveform with an artifact due to movement of the pressure transducer during the collection process

Movement causes large spikes in the recorded waveform as shown in Fig. 3. Other external factors can potentially interfere with the PVP measuring accuracy, such as adjusting the tubing or accidentally hitting the operative table [16].

### Acquiring porcine CVP

Porcine CVP waveforms were retrieved from a *Bleeding Detection Data Set* that was collected by researchers at Carnegie Mellon University and the Auton Lab at The Robotics Institute, and is available for public use [17]. The data set consists of vital signs measured at high frequency (250 Hz) using a bed-side hemodynamic monitoring system. Among the collected measurements are CVP and EKG waveforms from 52 healthy pigs subjected to slow bleeding. Each animal was sedated, instrumented, left to rest for half an hour, and then bled at the fixed rate of 20 mL/min. From the vital records, two 30-s samples are available—one sample from the resting period and one sample 2 min into the bleeding. The *Bleeding Detection Data Set* is available at mathieu.guillame-bert.com. The CVP and EKG waveforms were down sampled to 100 Hz to be consistent with the pediatric PVP waveforms for later analysis.

### Data cleaning algorithm

**Fig. 4:**
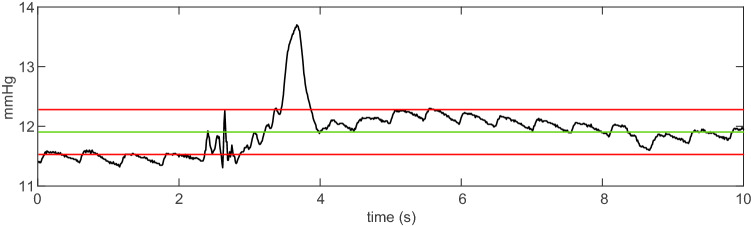
An illustration of the data pre-processing algorithm method. One-second of a PVP waveform is plotted and a red box with a red cross encloses an artifact that is to be removed. The green center line is the mean value of the waveform during the one second, and the red lines above and below the green line are the values one standard deviation above and below the mean, respectively

Due to waveform contamination from undesired artifacts mentioned previously, an algorithm was developed using MATLAB (*MathWorks Inc., Natick, Massachusetts, USA*) to pre-process the data and remove the unwanted sections of the waveforms. First, the entire PVP waveform was sampled at a rate of 100 Hz from LabChart 8 (*ADInstruments*) for each patient. After sampling the waveform, the PVP data was exported into a custom MATLAB program. For isoflurane patients, the corresponding MAC values were exported alongside the corresponding PVP waveforms. The algorithm takes sections of the PVP data at a user-selected length of time to analyze. For every section of the PVP waveform signal, the mean value of the data values in that section was calculated, and if any data points in that time section exceeds above or below the user-defined number of standard deviations, then the entire section of data is removed; this method is illustrated in Fig. 4. The algorithm goes through the entire PVP waveform, which can be up to 4 h long for the isoflurane patients, and removes sections of the data that contain spikes within the segments due to movement. The process takes a maximum of 2 min.

### Fast Fourier transform

After the cleaning algorithm, the data are divided into 10 second windows. Each window contains only a continuous waveform, i.e. if the cleaning algorithm removed any amount of data from a window then the entire window was discarded. Each window is converted to the frequency domain by means of Fast Fourier Transform (FFT). With a time-domain sampling rate of 100 Hz, the signal covers a frequency range of 50 Hz. Due to the low frequency of PVP signals, only the section of the signals within the frequency range from 0 to 20 Hz are used for further processing. Thus, each frequency domain window has a frequency range of 20 Hz. The frequency range was reduced to avoid unnecessary complexity in the statistical analysis [18].

### Clinical demographics

For the propofol testing, 39 patients were initially included in the study. Three patients were removed because a Nexiva catheter (*Beckon Dickinson Infusion Therapy Systems, Sandy, Utah, USA*) was used instead of the PIV catheter, resulting in a distinctly different PVP waveform [16]. Two other patients were discarded because their PIV catheters were inserted into the foot. Eleven patients were excluded due to either a flat PVP waveform due to incorrect zeroing of catheter or other circumstances that rendered the data unusable. These selections were performed in collaboration between all co-authors of this study. This resulted in a total of 23 patients used for waveform analysis. The patients were further sorted based on their hydrations status when they arrived at the emergency room, either hypovolemic with severe fluid loss, or euvolemic with normal fluid volume. Statistical testing for hypovolemic patients and euvolemic patients were conducted separately. For the isoflurane testing, nine patients were initially included in the study. Two patients were removed because the operation start time was not noted when LabChart began recording the PVP, making it difficult to relate MAC and PVP. The seven isoflurane patients were further sorted, based on the number of MAC groups used during the operation. For each patient, there were *n* MAC groups that were assigned a group number *n* > 0 when MAC fell between *n*-1 and *n*-0.1. For example, if MAC ranged between [0–0.9], then it would be classified as MAC group 1.

When testing the strength of the relationship between the arterial and venous circulations, all seven craniosynostosis EKG and PVP and 52 porcine EKG and CVP waveforms were used. Only five pyloric patients had simultaneously collected pulse and PVP waveforms to analyze.

### Statistical analysis: k-NN and MANOVA

Statistical analysis was performed with RStudio (*RStudio Inc., Boston, Massachusetts, USA*) and MATLAB. MATLAB was used to develop k-nearest neighbor (k-NN) statistical models and build machine learning prediction systems for the propofol and isoflurane PVP waveform.

RStudio was used to perform the multivariate analysis of variance (MANOVA) test. For MANOVA test, a significance level of 0.05 was used. The Pillai’s trace was the chosen test statistic due to its robustness [18]. For the propofol waveforms, the independent variable was the classification number that was assigned to the intraoperative and preoperative PVP signals and the dependent variable was the PVP waveform. For the isoflurane waveforms, the independent variable was the MAC group, and the dependent variable was the PVP waveform.

Pairwise MANOVA was also applied for all groups of data collected from both the propofol and isoflurane data to ensure the results were reliable and are shown in Supplementary Table 1.

### Statistical analysis: correlation coefficient between arterial and venous circulation

**Fig. 5:**
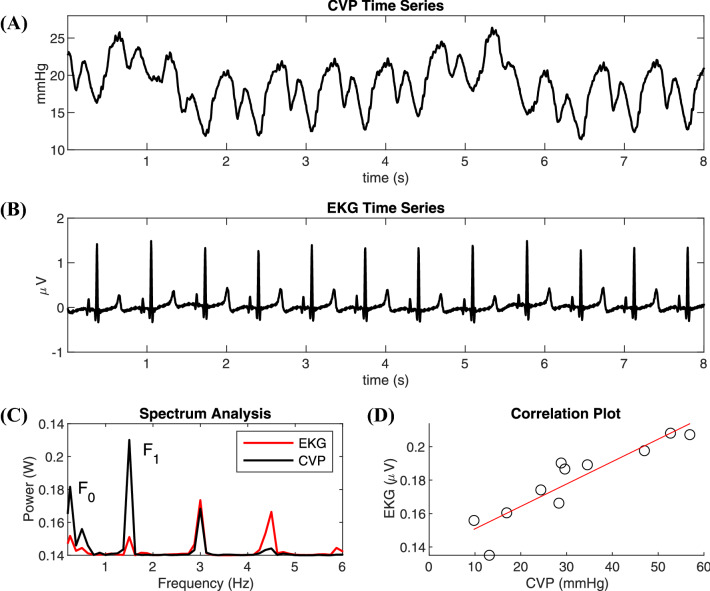
**a** Eight-second time series example of porcine central venous pressure (CVP) waveform before bleeding, (**b**) simultaneously recorded eight-second time series example of the porcine electrocardiography (EKG) waveform before bleeding, (**c**) power spectral density of the CVP and EKG with respiratory rate, F_0_, and pulse rate, F_1_, labeled, (**d**) correlation coefficient plot at F_1_. Eight-second examples were used for better illustration purposes in (**a**) and (**b**), but the actual snippet size used for the methods was two-seconds

Using MATLAB, the time series of the PVP and CVP waveforms along with their simultaneous EKG waveforms were sectioned into 2-s snippets, and an FFT was applied. For each snippet, the Power Spectral Density (PSD) was plotted and the magnitude of the amplitude of the frequencies F_0_, corresponding to the respiratory rate, and F_1_, corresponding to the pulse rate, were calculated. A time-domain sample of a CVP and the simultaneously recorded EKG waveform undergoing this step is illustrated in Fig. 5 along with the corresponding PSD and correlation coefficient scatter plot.

When using the craniosynostosis cohort, the same methods stated above were implemented, but each snippet was only affected by one MAC group, i.e. if a snippet was taken when the anesthetic dosage was increasing from MAC group 1 to MAC group 2 then that snippet was discarded. Further, instead of comparing correlation coefficients between the stages before and after anesthetic application, the craniosynostosis cohort was used to compare the correlation coefficients across each MAC group. It is important to note that the craniotomy length for each patient was significantly reduced after sections containing artifacts were removed. Therefore, not every MAC group had the sufficient amount of data points needed to compute the correlation coefficient.

The statistical test for Pearson’s correlation coefficient was calculated (Eq. 1) between the PVP/CVP and EKG waveforms at the F_0_ and F_1_ frequencies for each subject.

1 $$ {\rho}_{X,Y}=\frac{\sum \left(\left(X-{\mu}_X\right)\left(Y-{\mu}_Y\right)\right)}{\sigma_X{\sigma}_Y} $$

In the above equation, X is the magnitude of the amplitude at F_0_ or F_1_ from the PVP/CVP waveform and Y is the magnitude of the amplitude at F_0_ or F_1_ from the EKG waveform. The corresponding *p*-values were also recorded and a significance level of 0.05 was used.

## Results

### Patient demographics

The average weight of the 15 enrolled euvolemic pyloric stenosis pediatric patients was 4.14 kg (kg) with a standard deviation of 0.68 kg. The average weight of the eight hypovolemic patients was 3.70 kg with a standard deviation of 0.74 kg, which was lower than the euvolemic patients. After enrollment, fluids were given to the hypovolemic patients so that at the time of the operation, the 23 patients were all considered euvolemic. The average weight of the enrolled craniosynostosis pediatric patients was 10 kg with a standard deviation of 3.66 kg.

### MANOVA

The MANOVA *p* values and the Pillai’s trace were calculated and are shown in Tables 1 and 2. The results in the two tables show a significant relationship between the PVP signal and the effect of anesthetics.

**Table 1:** MANOVA results determining infused anesthetic effect

	df	df error	F	Partial η^2^	*p*-value
Hypovolemia	50	327	6.0	0.478	<0.01
Euvolemia	50	302	3.8	0.388	<0.01

**Table 2:** MANOVA results determining inhaled anesthetic effect

Patient #	df	df error	F	Partial η ^2^	*p*-value
3	50	231	4.6	0.499	<0.01
4	50	221	3.0	0.406	<0.01
5	50	249	2.8	0.359	<0.01
6	50	571	17.3	0.602	<0.01
7	50	101	2.9	0.586	<0.01
8	50	110	7.3	0.768	<0.01
9	50	226	3.7	0.450	<0.01

### K-Nearest neighbor

Prediction models were designed using k-nearest neighbor (k-NN) (k = 1) for creating the machine learning prediction systems for the propofol and isoflurane patients. For both the propofol and isoflurane studies, 70% of the data were used for training and the remaining 30% were used for testing. Results from the machine learning systems are shown in Tables 3 and 4 below, and their respective contingency matrices are available in Supplementary Tables 6 and 7 in the Supplementary.

**Table 3:** K-NN prediction results for infused anesthetic out of the total number of windows

	Correct prediction	Incorrect prediction
Hypovolemia	96/106	10/106
Euvolemia	102/114	12/114

**Table 4:** K-NN prediction results for inhaled anesthetic out of the total number of windows

Patient #	Correct prediction	Incorrect prediction
3	66/82	16/82
4	55/67	12/67
5	89/90	1/90
6	143/186	43/186
7	27/35	8/35
8	23/28	5/28
9	64/82	18/82

**Fig. 6:**
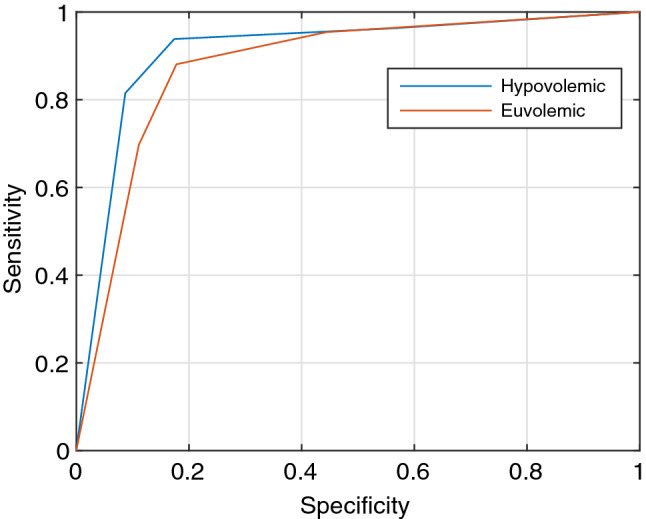
Receiver operating characteristic (ROC) curve for identification of the infused anesthetic, propofol, plotted as 1-specificity vs sensitivity

**Fig. 7:**
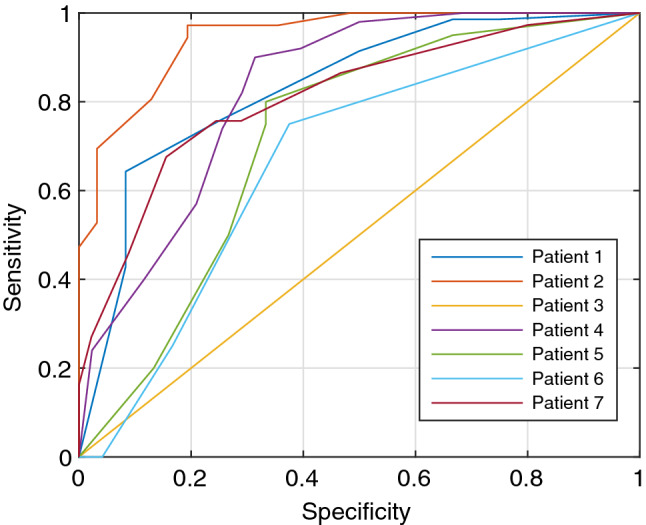
ROC curve for identification of the level of anesthetic inhaled as minimum alveolar concentration (MAC), plotted as 1-specificity vs sensitivity

Supplementary Tables 6 and 7 show the number of data points that the machine learning algorithm predicted correctly for the propofol and the isoflurane. A receiver operating characteristic (ROC) curve was plotted for each cohort to illustrate the machine learning model’s ability to classify the data and is shown in Figs. 6 and 7. ROC is plotted as 1-specificity vs sensitivity, with 1-*specificity = |FP|/(|FP| + |TN|)* and *sensitivity = |TP|/(|TP| + |FN|)* [13]*.* The values for false positive (FP), false negative (FN), true positive (TP), and true negative (TN) can be found in the contingency tables in Supplementary Tables 6 and 7.

**Fig. 8:**
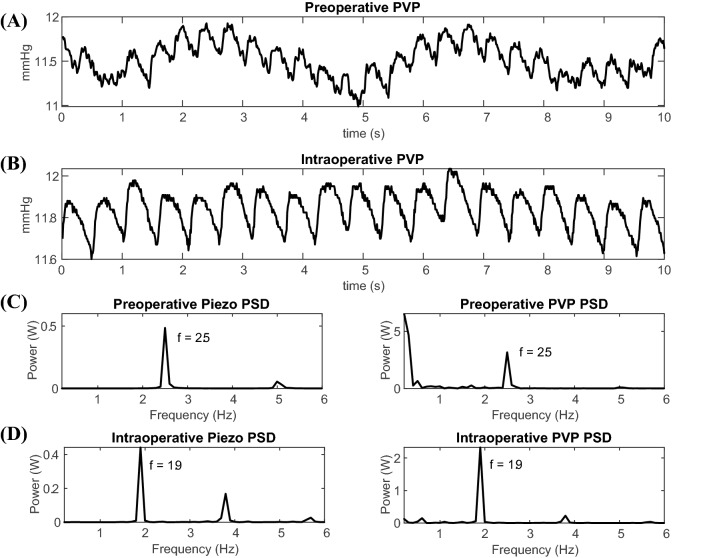
The time series PVP amplitude is lower when an infused anesthetic, propofol, was introduced and the PVP harmonics follow the piezoelectric. Euvolemic Patient 2’s PVP in time domain for preoperative, *before anesthetic*, (**a**) and intraoperative, *after anesthetic*, (**b**) waveforms. Power spectral densities of the PVP and piezoelectric waveforms are illustrated for the preoperative (**c**) and intraoperative (**d**) stages

The piezoelectric signal was measured along with the PVP to find if there is any correlation between the two signals. From Fig. 8a, b, it is clear that the two waveforms have harmonic peaks at similar frequencies. In Fig. 8c, the harmonic with the highest amplitude is at 2.5 Hz, which is higher than the frequency, 1.9 Hz, of the highest amplitude in Fig. 8d.

**Fig. 9:**
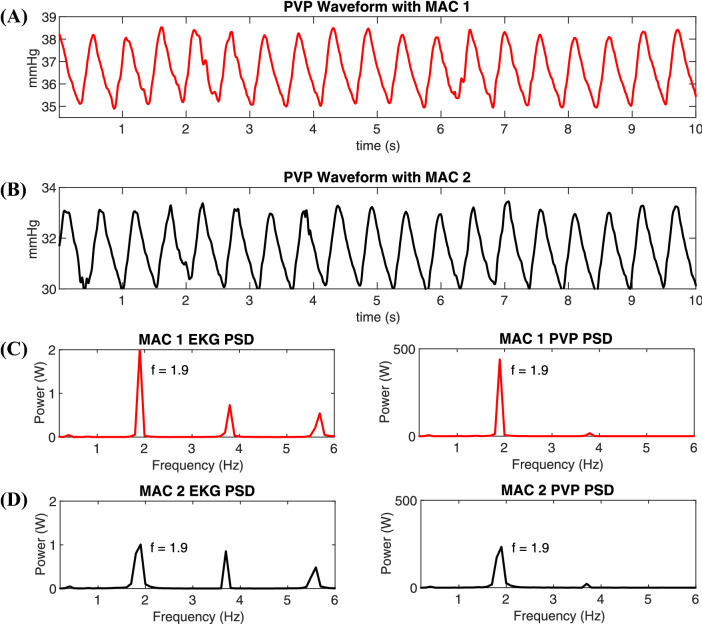
The time series PVP amplitude is lower in higher MAC dosages and the PVP harmonics follow the electrocardiography (EKG). Craniosynostosis cohort Patient 4’s PVP time-domain waveform in MAC group 1 (**a**) and MAC group 2 (**b**). Power spectral densities of the PVP and EKG waveforms are illustrated for MAC group 1 (**c**) and MAC group 2 (**d**)

The EKG was measured along with the PVP to find if there is any correlation between the two waveforms. In Fig. 9a, b, the two signals have harmonic peaks at similar frequencies. In Fig. 9c, the harmonic with the highest amplitude is at 1.9 Hz, which is similar to the frequency of the highest amplitude in Fig. 9d.

### Correlation coefficient between arterial and venous circulation

Due to the large number (over one hundred) of correlation coefficients calculated, each coefficient and corresponding *p* value is not listed individually in this section. Instead the results are summarized by each stage for each cohort. All correlation coefficient results for each cohort can be found in Supplementary Tables 8 and 9.

### Pyloric cohort

In the pyloric cohort before anesthetics, the average F_0_ was 0.24 Hz (~14 breaths per minute) and the average F_1_ was 2.23 Hz (~133 bpm). Only two of the five pediatric patients had a correlation coefficient at F_0_ with a respective *p* value below 0.05 before the anesthetic was applied, and three had coefficients with *p* values lower than 0.05 at F_1_. The strongest correlation coefficient at frequency F_0_ was 0.39 and the weakest at 0.35. The strongest correlation coefficient at frequency F_1_ was 0.96 and the weakest 0.13.

In the pyloric cohort after anesthetics, the average F_0_ was 0.25 Hz (~15 breaths per minute) and the average F_1_ was 2.62 Hz (~157 bpm). Only one of the five pediatric patients had a correlation coefficient at F_0_ with a respective *p* value below 0.05 after anesthetic application, and four had correlation coefficients with *p* values lower than 0.05 at F_1_. The coefficient at F_0_ was 0.46. The strongest coefficient at F_1_ was 0.96 and the weakest 0.57.

### Craniosynostosis cohort

In the craniosynostosis cohort, there were only two patients that had sufficiently large enough data under at least two different MAC groups, thus only their results will be stated. All of the results from the craniosynostosis cohort are available in Supplementary Tables 10–13.

Patient 5 had correlation coefficients computed for MAC groups 3 and 4. In MAC group 3, the F_0_ was 0.34 Hz (~21 breaths per minute). The correlation coefficient at F_0_ was −0.084 and the *p* value >0.05. Also, in MAC group 3, the F_1_ was 2.04 Hz (~123 bpm), and the correlation at that frequency was 0.395 with a *p* value>0.05. In MAC group 4, the F_0_ was 0.21 Hz (~13 breaths per minute) with a correlation of −0.004 and *p* value >0.05. Also in MAC group 4, the F_1_ was 1.85 Hz (~111 bpm) with a correlation of 0.036 with a *p* value <0.05.

Patient 7 had correlation coefficients computed for MAC groups 2, 3, and 4. In MAC group 2, the F_0_ was 0.40 Hz (~24 breaths per minute) with a correlation coefficient of −0.115 and *p* value >0.05. The F_1_ was 1.89 Hz (~114 bpm) with a correlation coefficient of 0.003 and *p* value >0.05. In MAC group 3, the F_0_ was 0.40 Hz (~24 breaths per minute) with a correlation coefficient of 0.128 and *p* value >0.05. The F_1_ was 1.85 Hz (~ 111 bpm) with a correlation coefficient of 0.360 and *p* value >0.05. In MAC group 4, the F_0_ was 0.22 Hz (~14 breaths per minute) with a correlation coefficient of 0.001 and *p* value >0.05. The F_1_ was 1.78 Hz (~ 107 bpm) with a correlation coefficient of 0.052 and *p* value >0.05.

### Porcine cohort

In the porcine cohort before bleeding, the average F_0_ was 0.21 Hz (~13 breaths per minute) and the average F_1_ was 1.51 Hz (~91 bpm). Out of the 52 pigs before bleeding, 22 had correlation coefficients with a *p* value below 0.05 at frequency F_1_. The strongest coefficient was 0.93 and the weakest 0.53. At F_0_ before bleeding, 33 pigs had coefficients with a *p* value below 0.05, with the strongest being 0.95 and the weakest being 0.53.

In the porcine cohort during bleeding, the average F_0_ was 0.21 Hz (~13 breaths per minute) and the average F_1_ was 1.47 Hz (~88 bpm). After bleeding, 21 of the 52 pigs had correlation coefficients with a *p* value below 0.05 at frequency F_1_. The strongest coefficient was 0.90 and the weakest 0.52. At frequency F_0_, 33 pigs had coefficients with a *p* value below 0.05, with the strongest being 0.94 and the weakest being 0.54.

## Discussion

PVP can be measured via a peripheral IV in the extremities, making it easier to access and measure compared to central venous pressure (CVP). CVP is traditionally used in assessing the overall circulatory status of a patient in an intensive care or operative setting, and to guide resuscitation [19]. Several studies have shown that CVP and PVP correlate significantly [19–21]. The need for a less invasive method of measuring volume status inspired the use of PVP waveforms.

The venous system is highly compliant and can accommodate large changes in volume with minimal changes in pressure. However, the detection of the subtle changes in PVP waveforms as a result of volume loss is made possible due to signal amplifying technologies that can extract hemodynamic signals in the frequency domain through Fast Fourier Transformation (FFT) [22, 23]. The frequency domain PVP signals can then be analyzed with advanced statistical and machine learning algorithms. Venous waves are generated by the cardiac cycle and propagated as harmonics [7]. The f1 waveform which correlates with the heart rate, has already been shown to be affected already by very mild hypovolemia [9]. Previous work in animal models has demonstrated that FFT of a PVP waveform correlated with volume status is more sensitive than standard vital sign monitoring [8]. Despite the robust evidence of the correlation between PVP waveforms and volume status, both the exact mechanism behind this link, and potential confounding parameters have not been thoroughly investigated.

Our studies have discovered a significant relationship between both the infused and inhaled anesthetics and the PVP waveform. More specifically, when propofol is administered, the PVP amplitude of the intraoperative waveform decreases compared to the amplitude of the preoperative waveform. The relationship between propofol and PVP is illustrated in Fig. 8. After administering an infused anesthetic, the PVP amplitude directly decreases. Our results find that the piezoelectric and PVP frequencies correlate, showing that pulse rate decreases when the patient is under anesthetics. For the isoflurane patients, whenever MAC increases, the PVP waveform decreases. This demonstrates that increasing MAC immediately dilates the veins and reduces venous pressure; the relationship is illustrated in Fig. 9a, b. The EKG was measured along with the PVP to find the frequency that corresponds to the heart rate and weather it is matching the frequency at the highest peak of the PVP waveform. Our results show a robust mimicking between the frequency of PVP and the frequency of EKG and the frequencies at the highest amplitude in Fig. 9c, d are equal, 1.9 Hz.

In addition to identifying a relationship between PVP waveforms and anesthetics, we were also able to demonstrate that a machine learning prediction model can distinguish between PVP waveforms that are influenced by propofol and those with no anesthetics present with at least 89% accuracy, as displayed in Table 3. The ROC curve in Fig. 6 has a high area under the curve for both the hypovolemic and euvolemic data, which illustrates a high-performance measure for the machine learning model [24]. The machine learning prediction model for the isoflurane patients was able to accurately distinguish between the MAC groups in each patient’s PVP waveform at least 77% of the time, shown in Table 4. The ROC curve in Fig. 7 shows the highest area under the curve for patient 4, so the model has the best performance for that patient. The curves for patients 1, 4, 5, 6, and 7 show that the model is performing well at predicting the MAC groups but fails to perform for Patient 3. This may be due to Patient 3 having a smaller amount of clean PVP data to analyze or insufficient training data for each of the MAC groups specific to the patient. Overall, these high correct prediction results further support the conclusion that anesthetics affect the PVP waveform.

Our results further show that once an infused anesthetic is applied, the relationship between arterial and venous circulation becomes stronger, likely due to the dilation of the vein and subsequent decrease in distance between the vein and neighboring artery. Once these vessels are in close proximity to each other, the pulse from the artery is reflected in the vein, and our results support this claim with the strongly positive correlation coefficients between the venous pressure and EKG waveforms. The results suggest that the relationship is strong regardless of whether or not the venous pressure is specifically PVP or CVP. More specifically, the relationship between the central venous and arterial pressure is equally strong before and during blood loss. The relationship between the peripheral venous and arterial pressure may become stronger once an inhaled anesthetic has been applied, but these results are inconclusive in supporting or denying that claim.

It is important to note that the results of this study were reached independently amongst three different cohorts. Knowing that anesthetics affect both the venous pressure waveforms and cross-talk between peripheral and arterial pressure in each cohort only strengthens the final conclusion. Even though there were slight differences in each cohort, e.g. infused vs inhaled anesthetics or PVP vs CVP, they led to the same results which indicate that anesthetics are playing a large role in venous waveforms, and that needs to be taken into account when future researchers utilize those waveforms.

**Fig. 10:**
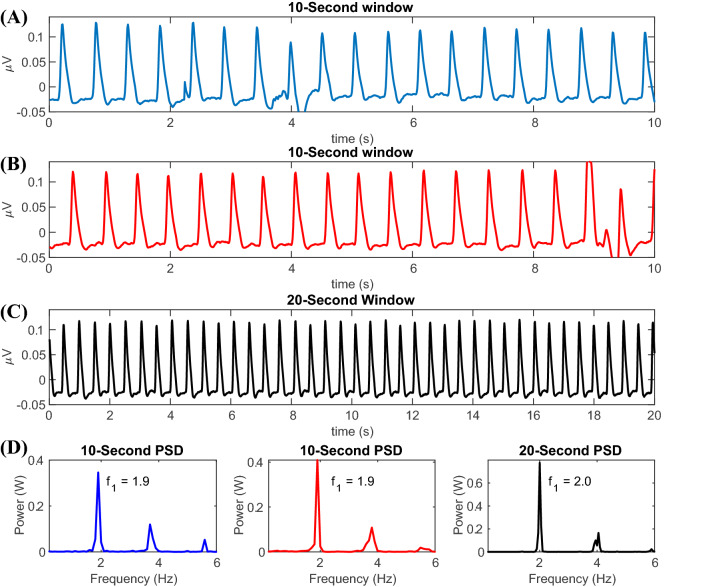
(**a** and **b**) Two separate, non-overlapping 10-s windows of the PVP waveform. In each of the windows, the heart rate is approximately 1.90 bpm, (**c**) a separate 20-s window of the PVP waveform in the same patient and the heart rate is approximately 2.00 bpm, (**d**) the Fast Fourier Transform (FFT) of each of the 10- and 20-s windows where the color of the line corresponds to the respective signal from (**a**), (**b**), and (**c**). The heart rate frequencies are aligned at 1.9 Hz for the 10-s windows and the FFT of the 20-s window shows a heart rate frequency at 2.0 Hz

In future studies, it would be beneficial to compare these results with an analysis that utilizes an amplitude density instead of a power spectral density to determine if the peak amplitudes corresponding to the heart rate and respiratory rate frequencies are comparable to the peak power at the same frequencies. When calculating the amplitude or power spectral densities, it is possible to use a different window size when calculating the FFT. The choice of a 10-s window is a tradeoff between frequency domain resolution and the stationarity of the signal. A 10-s window results in a frequency domain resolution of 0.1 Hz, which is sufficient to identify finer changes in the frequency domain. In addition, a smaller window can ensure that the signals are approximately stationary within one window. If the window size is too long, it is possible that the signals within a window are no longer stationary, that is, the statistical properties of the signal might change within a window, and this will negatively affect the accuracy of the frequency-domain analysis. As an example, it is shown in Fig. 10 that there is a shift in heart rate frequency caused by increasing the window size from 10 s to 20 s.

Another aspect that should be considered in the future is the Systemic Vascular Resistance (SVR). The SVR would help to assess whether the vasodilation of the vessels are dampening the pulse pressure waveform and power at the heart rate and respiratory rate frequencies. The SVR calculation could be reported along with the correlation value to provide a more precise explanation of what is occurring during the cross-talk between the venous and arterial circulations.

### Limitations

This study focused on a small cohort of patients when determining the effect of anesthetics in the craniosynostosis cohort, and for calculating the strength of the relationship between the peripheral arterial and venous circulations in the pediatric data. For future analysis, more pediatric patients will be enrolled in the study. In this study, only two craniosynostosis patients had sufficiently long waveforms under at least two different anesthetic dosages, so it is vital to test an inhaled dataset before claiming a relationship between arterial and venous circulations under the effect of an inhaled anesthetic. Increasing patient numbers will give a better understanding of the effect of anesthetics and enhance the robustness of the results. This study cannot prove causality, as there could be confounding factors in the clinical parameters of these patients that affected the anesthesia and PVP waveform. However, this is a preliminary study that indicates that there is a correlation between anesthetics and PVP, as well as moderate to strong relationship between the venous and arterial circulations in these cohorts. We encourage future PVP waveform researchers to consider that anesthetics may be affecting the waveforms, and to also account for the cross-talk present between the venous and arterial circulations.

## Conclusion

Propofol and isoflurane are known potent vasodilators, and our results indicate that the subsequent changes in vascular resistance are reflected in the venous circulation and thus PVP signal as well. Our results also suggest that there is a relationship between the venous and arterial circulations, and that pulse may affect PVP and CVP signals that are collected in cases of dehydration, hemorrhage, and anesthetics. Future research in PVP waveforms should take into account any anesthetic agents the patient has received and the heart rate of the patient. This clear relationship should be considered in any future application of PVP signal technology.

## Electronic supplementary material

Below is the link to the electronic supplementary material.

Electronic supplementary material 1 (PDF 221 kb)

Electronic supplementary material 2 (PDF 73 kb)

Electronic supplementary material 3 (PDF 157 kb)

Electronic supplementary material 4 (PDF 210 kb)

Electronic supplementary material 5 (PDF 314 kb)

## Data Availability

The pediatric datasets used in this study can be obtained by contacting the corresponding author. The porcine dataset is publicly available for use at mathieu.guillame-bert.com, as well as the paper regarding this dataset by Guillame-Bert (2017).
