# Supplementary material for: Anesthetics affect peripheral venous pressure waveforms and the cross-talk with arterial pressure
Source: J Clin Monit Comput. 2021 Feb 19;36(1):147–59. doi: 10.1007/s10877-020-00632-6 (PMC8894218; doi:10.1007/s10877-020-00632-6)
Supplement: Supplementary file 1 — Electronic supplementary material 1 (PDF 221 kb) [file 10877_2020_632_MOESM1_ESM.pdf]

Supplementary Material

Article Title: Anesthetics Affect Peripheral Venous Pressure Waveforms and the Cross-Talk with Arterial Pressure

Authors: Ali Z. Al-Alawi[1]*, Kaylee R. Henry[1]*, Lauren D. Crimmins[1], Patrick C. Bonasso[3], Md Abul Hayat[2], Melvin S. Dassinger[3], Jeffrey M. Burford[3], Hanna K. Jensen[1], Joseph Sanford[4], Jingxian Wu[2], Kevin W. Sexton[5], Morten O. Jensen[1]

From the:

[1]Department of Biomedical Engineering, University of Arkansas, Fayetteville, AR, USA,

[2]Department of Electrical Engineering, University of Arkansas, Fayetteville, AR, USA,

[3]Division of Pediatric Surgery, University of Arkansas for Medical Sciences, Little Rock, AR, USA,

[4]Department of Anesthesiology, University of Arkansas for Medical Sciences, Little Rock, AR, USA.

[5]Department of Surgery, University of Arkansas for Medical Sciences, Little Rock, AR, USA.

[*] These authors contributed equally to this work.

Corresponding Author: Morten Jensen

Department of Biomedical Engineering

University of Arkansas

White Hall Engineering

790 W Dickson Street

Fayetteville, AR 72701

Tel. 479-575-4997

mojensen@uark.edu

Tables 1 – 5: MANOVA pairwise of isoflurane patients

Group 1 → [0-0.9]

Group 2 → [1-1.9]

Group 3 → [2-2.9]

Group 4 → [3-3.9]

Patient 1

	Group 1	Group 2
Group 2	0.02	-
Group 3	0.02	NA*

Patient 2

	Group 1	Group 2
Group 2	0.02	-
Group 3	0.02	0.02

Patient 5

	Group 1	Group 2
Group 2	0.029	-
Group 3	0.029	0.255

Patient 6

	Group 1	Group 2	Group 3
Group 2	0.02	-	-
Group 3	0.02	0.02	-
Group 4	0.02	0.02	NA*

Patient 7

	Group 1	Group 2	Group 3
Group 2	0.029	-	-
Group 3	0.02	0.137	-
Group 4	0.029	0.039	NA*

*NA → No pairwise calculated because the number of windows is less than the required number of required variables, 50
