# Supplementary material for: Anesthetics affect peripheral venous pressure waveforms and the cross-talk with arterial pressure
Source: J Clin Monit Comput. 2021 Feb 19;36(1):147–59. doi: 10.1007/s10877-020-00632-6 (PMC8894218; doi:10.1007/s10877-020-00632-6)
Supplement: Supplementary file 2 — Electronic supplementary material 2 (PDF 73 kb) [file 10877_2020_632_MOESM2_ESM.pdf]

Tables 6 – 7: Contingency tables for propofol and isoflurane patients, respectively

	Testing data			Training Data		
Euvolemic		Preop	OR		Preop	OR
	Preop	96	13	Preop	244	11
	OR	8	37	OR	25	80
	Testing data			Training Data		
Hypovolemic		Preop	OR		Preop	OR
	Preop	78	3	Preop	115	3
	OR	3	20	OR	11	43

Patient #	Testing data			Training Data		
1		MAC 1	MAC 2		MAC 1	MAC 2
	MAC 1	60	10	MAC 1	164	0
	MAC 2	6	6	MAC 2	0	29
2		MAC 1	MAC 2		MAC 1	MAC 2
	MAC 1	28	8	Group 1	83	0
	MAC 2	4	27	Group 2	0	74
3		MAC 1	MAC 2		MAC 1	MAC 2
	MAC 1	0	1	MAC 1	1	0
	MAC 2	0	89	MAC 2	0	209
4		MAC 1	MAC 2		MAC 1	MAC 2
	MAC 1	97	3	MAC 1	234	0
	MAC 2	8	78	MAC 2	0	202
5		MAC 1	MAC 2		MAC 1	MAC 2
	MAC 1	17	3	MAC 1	48	0
	MAC 2	5	10	MAC 2	0	35
6		MAC 1	MAC 2		MAC 1	MAC 2
	MAC 1	1	3	MAC 1	10	0
	MAC 2	2	22	MAC 2	0	57
7		MAC 1	MAC 2		MAC 1	MAC 2
	MAC 1	25	12	MAC 1	87	0
	MAC 2	6	39	MAC 2	0	104
