# Supplementary material for: Anesthetics affect peripheral venous pressure waveforms and the cross-talk with arterial pressure
Source: J Clin Monit Comput. 2021 Feb 19;36(1):147–59. doi: 10.1007/s10877-020-00632-6 (PMC8894218; doi:10.1007/s10877-020-00632-6)
Supplement: Supplementary file 3 — Electronic supplementary material 3 (PDF 157 kb) [file 10877_2020_632_MOESM3_ESM.pdf]

Tables 8 – 9: Correlation coefficients and p-values for the pyloric cohort before and after anesthetic, respectively

Before Anesthetic						
	F_1	F_1 ρ	p-value	F_0	F_0 ρ	p-value
1*	-	-	-	-	-	-
2	2.50	0.96	0.015	0.22	0.056	0.742
3	2.17	0.42	0.029	0.23	0.152	0.381
4	1.67	0.13	0.643	0.25	0.352	0.000
5	2.50	-0.03	0.504	0.26	0.393	0.016

After Anesthetic						
	F_1	F_1 ρ	p-value	F_0	F_0 ρ	p-value
1*	-	-	-	-	-	-
2	2.50	0.96	0.019	0.25	-0.146	0.425
3	2.67	0.64	0.016	0.25	0.112	0.602
4	2.50	0.57	0.608	0.25	0.460	0.014
5	2.50	0.61	0.088	0.25	0.110	0.626

*As the first patient recorded using these methods, Patient 1 was not included in the correlation coefficient analysis due to inconsistencies in the signal recording compared to the later acquired patients.

Tables 10 – 13: Correlation coefficients and p-values for the craniosynostosis cohort for MAC group 1, MAC group 2, and MAC group 3, respectively. As stated previously, it is important to note that the craniotomy length for each patient was significantly reduced after sections containing artifacts were removed. Therefore, not every MAC group had the sufficient amount of points needed to compute the correlation coefficient. The MAC groups that had a sufficient amount of data for each patient are:

Patient 1 → MAC group 1

Patient 2 → MAC group 1

Patient 3 → MAC group 1

Patient 4 → MAC group 1

Patient 5 → MAC groups 3 and 4

Patient 6 → MAC group 1

Patient 7 → MAC groups 2, 3, and 4
