# Supplementary material for: Anesthetics affect peripheral venous pressure waveforms and the cross-talk with arterial pressure
Source: J Clin Monit Comput. 2021 Feb 19;36(1):147–59. doi: 10.1007/s10877-020-00632-6 (PMC8894218; doi:10.1007/s10877-020-00632-6)
Supplement: Supplementary file 5 — Electronic supplementary material 5 (PDF 314 kb) [file 10877_2020_632_MOESM5_ESM.pdf]

Tables 14 – 15: Correlation coefficients and p-values for the porcine cohort before and during bleeding, respectively

Before Bleeding						
Pig #	F_1	F_1 ρ	p-value	F_0	F_0 ρ	p-value
1	1.5	0.500	0.057	0.23	0.838	0.000
2	1.4	0.256	0.357	0.23	0.538	0.038
3	1.5	0.403	0.136	0.23	0.844	0.000
4	1.4	0.805	0.000	0.23	0.951	0.000
5	1.63	-0.084	0.7673	0.22	0.612	0.015
6	1.4	0.622	0.013	0.22	0.659	0.008
7	1.63	-0.532	0.041	0.23	0.942	0.000
8	1.43	0.226	0.418	0.22	0.786	0.000
9	1.46	0.267	0.337	0.23	0.846	0.000
10	1.37	-0.181	0.519	0.22	0.662	0.007
11	1.27	0.254	0.361	0.22	0.719	0.002
12	1.46	0.879	0.000	0.28	0.714	0.003
13	1.63	0.543	0.037	0.20	0.792	0.000
14	1.77	0.890	0.000	0.25	0.861	0.000
15	1.33	0.767	0.000	0.20	0.533	0.041
16	1.27	0.571	0.026	0.23	0.806	0.000
17	1.5	0.295	0.286	0.22	0.802	0.000
18	2.3	0.451	0.092	0.20	0.784	0.000
19	1.63	0.641	0.010	0.22	0.748	0.001
20	1.43	0.356	0.193	0.22	0.948	0.000
21	1.43	0.760	0.001	0.22	0.835	0.000
22	1.37	.096	0.733	0.25	0.258	0.354
23	2.17	0.412	0.127	0.22	0.431	0.108
24	1.7	0.597	0.019	0.18	0.801	0.000
25	1.37	0.709	0.003	0.20	0.875	0.000
26	1.5	0.075	0.791	0.20	-0.159	0.573
27	1.43	0.525	0.045	0.20	0.733	0.002
28	1.37	0.541	0.037	0.23	0.509	0.053
29	1.3	-0.007	0.981	0.20	0.730	0.002
30	1.57	-0.363	0.184	0.20	0.898	0.000
31	1.3	0.508	0.053	0.20	-0.312	0.258
32	2	0.472	0.078	0.23	-0.025	0.928
33	1.3	0.385	0.157	0.2	0.903	0.000
34	1.1	-0.410	0.129	0.2	0.065	0.818
35	1.43	0.387	0.154	0.22	0.119	0.672
36	1.3	0.687	0.005	0.20	0.493	0.062
37	1.5	-0.327	0.234	0.23	0.028	0.315
38	1.6	0.934	0.000	0.20	-0.009	0.974
39	1.37	0.664	0.007	0.20	0.799	0.000
40	1.3	-0.258	0.354	0.20	0.155	0.581
41	1.5	-0.200	0.474	0.20	0.549	0.034
42	1.6	-0.115	0.684	0.22	-0.161	0.567
43	1.3	-0.437	0.104	0.20	0.378	0.165
44	1.7	0.615	0.015	0.20	0.846	0.000
45	1.27	0.790	0.001	0.20	0.326	0.236
46	1.57	0.640	0.010	0.18	0.078	0.734
47	1.6	-0.334	0.224	0.20	0.910	0.000
48	1.77	-0.308	0.265	0.20	-0.264	0.337

49	1.5	-0.089	0.753	0.22	0.794	0.000
50	1.73	0.122	0.664	0.22	0.665	0.007
51	1.27	0.639	0.010	0.22	0.415	0.123
52	1.5	0.900	0.000	0.20	0.183	0.515

During Bleeding						
Pig #	F_1	F_1 ρ	p-value	F_0	F_0 ρ	p-value
1	1.50	-0.045	0.873	0.23	-0.007	0.981
2	1.40	-0.097	0.731	0.23	0.165	0.558
3	1.57	0.139	0.620	0.23	0.541	0.037
4	1.40	0.689	0.005	0.23	0.866	0.000
5	1.63	0.253	0.363	0.23	0.863	0.000
6	1.40	0.745	0.001	0.23	0.442	0.099
7	1.63	0.365	0.182	0.23	0.859	0.000
8	1.43	0.309	0.262	0.23	0.692	0.004
9	1.47	0.135	0.630	0.23	0.837	0.000
10	1.37	-0.712	0.003	0.23	0.789	0.000
11	1.27	0.833	0.000	0.23	0.248	0.374
12	1.47	-0.350	0.201	0.20	0.756	0.001
13	1.63	0.296	0.285	0.20	0.539	0.038
14	1.77	0.673	0.006	0.17	0.715	0.003
15	1.33	0.714	0.003	0.20	0.653	0.008
16	1.27	0.218	0.435	0.23	0.741	0.002
17	1.47	0.767	0.001	0.20	0.650	0.009
18	1.40	0.528	0.043	0.20	0.713	0.003
19	1.53	0.672	0.006	0.23	0.726	0.002
20	1.40	0.690	0.004	0.20	0.841	0.000
21	1.40	0.597	0.019	0.20	0.635	0.011
22	1.37	0.519	0.048	0.17	0.610	0.016
23	1.07	-	-	0.20	-0.081	0.776
24	1.67	0.811	0.002	0.20	0.927	0.000
25	1.33	0.904	0.000	0.20	0.485	0.067
26	1.47	0.294	0.287	0.20	0.911	0.000
27	1.43	0.643	0.009	0.20	0.627	0.012
28	1.33	0.364	0.182	0.23	0.631	0.011
29	1.27	0.441	0.100	0.20	0.637	0.011
30	1.60	-0.308	0.265	0.20	0.871	0.000
31	1.27	0.396	0.144	0.20	-0.015	0.960
32	2.00	0.147	0.601	-	-	-
33	2.20	0.436	0.104	0.20	0.92	0.00
34	1.10	-0.229	0.425	0.20	-0.149	0.596
35	1.40	0.453	0.090	0.20	0.184	0.511
36	1.27	0.206	0.463	0.20	0.266	0.339
37	1.53	0.865	0.000	0.20	0.565	0.028
38	1.63	0.769	0.001	0.20	-0.104	0.713
39	1.33	0.822	0.000	0.20	0.851	0.000
40	1.33	-0.124	0.659	0.20	0.468	0.078
41	1.5	-0.514	0.050	0.20	0.903	0.000
42	1.57	-0.071	0.802	0.23	0.866	0.000
43	1.27	0.304	0.270	0.20	0.481	0.069
44	1.60	0.799	0.000	0.20	0.838	0.000

45	1.27	0.319	0.247	0.20	0.005	0.986
46	1.53	0.218	0.435	0.17	0.403	0.137
47	1.53	0.511	0.052	0.20	0.937	0.000
48	1.77	0.723	0.002	0.20	-0.111	0.695
49	1.47	0.167	0.551	0.20	0.679	0.005
50	1.70	0.257	0.355	0.20	0.817	0.000
51	1.27	0.276	0.319	0.20	0.362	0.185
52	1.5	0.265	0.340	0.20	0.236	0.396
